# Quality of life and mental health measurements among patients with type 2 diabetes mellitus: a systematic review

**DOI:** 10.1186/s12955-023-02111-3

**Published:** 2023-03-22

**Authors:** Owiss Alzahrani, John P. Fletcher, Kerry Hitos

**Affiliations:** 1 Westmead Research Centre for Evaluation of Surgical Outcomes, Department of Surgery, Westmead Hospital, Sydney, Australia; 2 The University of Sydney, Faculty of Medicine and Health, Westmead Clinical School, Sydney, Australia; 3 Department of Community Medicine, Faculty of Medicine, King Abdulaziz University, Jeddah, Saudi Arabia

**Keywords:** Type 2 Diabetes Mellitus, Quality of life, Mental health, Systematic review, Questionnaires

## Abstract

**Background:**

Over the past few decades the benefits of assessing Quality of Life (QoL) and mental health in patients with Type 2 Diabetes Mellitus (T2DM) have steadily increased with limited studies relating to the most useful method to assess these patients. This study aims to identify, review, summarise, and evaluate the methodological quality for the most validated commonly used health-related QoL and mental health assessment measurements in diabetic patients.

**Methods:**

All original articles published on PubMed, MedLine, OVID, The Cochrane Register, Web of Science Conference Proceedings and Scopus databases were systematically reviewed between 2011 and 2022. A search strategy was developed for each database using all possible combinations of the following keywords: “type 2 diabetes mellitus”, “quality of life”, mental health”, and “questionnaires”. Studies conducted on patients with T2DM of ≥ 18 years with or without other clinical illnesses were included. Articles designed as a literature or systematic review conducted on either children or adolescents, healthy adults and/or with a small sample size were excluded.

**Results:**

A total of 489 articles were identified in all of the electronic medical databases. Of these articles, 40 were shown to meet our eligibility criteria to be included in this systematic review. Approximately, 60% of these studies were cross-sectional, 22.5% were clinical trials, and 17.5% of cohort studies. The top commonly used QoL measurements are the SF-12 identified in 19 studies, the SF-36, included in 16 studies, and the EuroQoL EQ-5D, found in 8 studies. Fifteen (37.5%) studies used only one questionnaire, while the remaining reviewed (62.5%) used more than one questionnaire. Finally, the majority (90%) of studies reported using self-administered questionnaires and only 4 used interviewer mode of administration.

**Conclusion:**

Our evidence highlights that the commonly used questionnaire to evaluate the QoL and mental health is the SF-12 followed by SF-36. Both of these questionnaires are validated, reliable and supported in different languages. Moreover, using single or combined questionnaires as well as the mode of administration depends on the clinical research question and aim of the study.

## Introduction

Over the last few decades, the increasing recognition of the impact of Type 2 Diabetes Mellitus (T2DM) on Quality of Life (QoL), mental health and overall physical and psychological health along with their useful measurement instruments has been well addressed in scientific literature [1]. The benefits of evaluating QoL and mental health in patients with T2DM have been appreciated. This includes the evaluation of the burden of the disease and its complications, which may contribute to the development of the most appropriate management and treatment plans in these vulnerable patient groups [2].

Moreover, physicians caring for patients with comorbid chronic illnesses that affect their QoL and mental health, such as T2DM, need to prioritise their diabetes management to ensure better care with the aim to focus on how healthcare systems influence these decisions [3]. This includes the stability of these decisions over time, with continuous surveillance based on proper and validated measurements [3–6].

Overall, the nature of QoL is complex and multidimensional with a variation in tools used between studies. The Australian Centre for Quality of Life’s directory of instruments reflects this further where there are more than 1000 variables included and although these intend to measure QoL each contains a variety of dependent variables [7]. Findings from other studies have linked the wrong measure to the concept of interest and there are numerous occasions where incorrect or different tools have been used or where their data is misinterpreted as QoL [8, 9]. Moreover, this will emphasise the importance of selecting an ideal reliable and valid measure that is useful to use throughout different cultures. Also, it should include a broad range of potentially independent domains covering all critical aspects of QoL [10].

Furthermore, the assessment of mental health in patients with diabetes requires multiple transitions geographically and socially. In addition, there is a need to identify patients lacking medical follow-up and are therefore, at increasing risk of poor mental health status including psychosocial problems such as depression, diabetes-emotional distress, anxiety, eating disorders, and cognitive impairment [11]. Hence, it is essential for clinicians to use a standardised tool that is of dynamic construct that incorporates comprehensiveness, sensitivity, and balance relative to subjectivity and brevity to help identify gaps and monitor psychological well-being and care among adult patients with T2DM. However, to date, measuring QoL and mental health outcomes in these patients remains a challenge and there are limited studies evaluating the quality of these tools.

Therefore, the aim of this systematic review is to identify, summarise, and evaluate the methodological quality for the most commonly used and validated health-related QoL and mental health assessment measurements in patients with T2DM.

## Methodology

The Systematic review was conducted on QoL, and mental health surveys published in PubMed, MedLine, OVID, The Cochrane Register, Web of Science Conference Proceedings and Scopus databases between the 1^st^ of January 2011 and the 31^st^ of July 2022. In addition, reference lists of the included studies and previous reviews on the topic were hand searched for potentially relevant studies. Search terms for each database included ‘type 2 diabetes mellitus’, ‘quality of life’, ‘mental health’, and ‘questionnaires’. No language restrictions were applied. We performed a systematic search in accordance with the *Preferred reporting items for systematic review and meta-analyses protocols (PRISMA) statement 2020* [12]. Our formulated research question was based on Participants, Concept, and Context (PCC) on ‘What is the most recent validated and commonly used measurement or questionnaire to assess the quality of life and mental health among adult diabetic patients in different languages?’.

### Inclusion and exclusion criteria

**Fig. 1:**
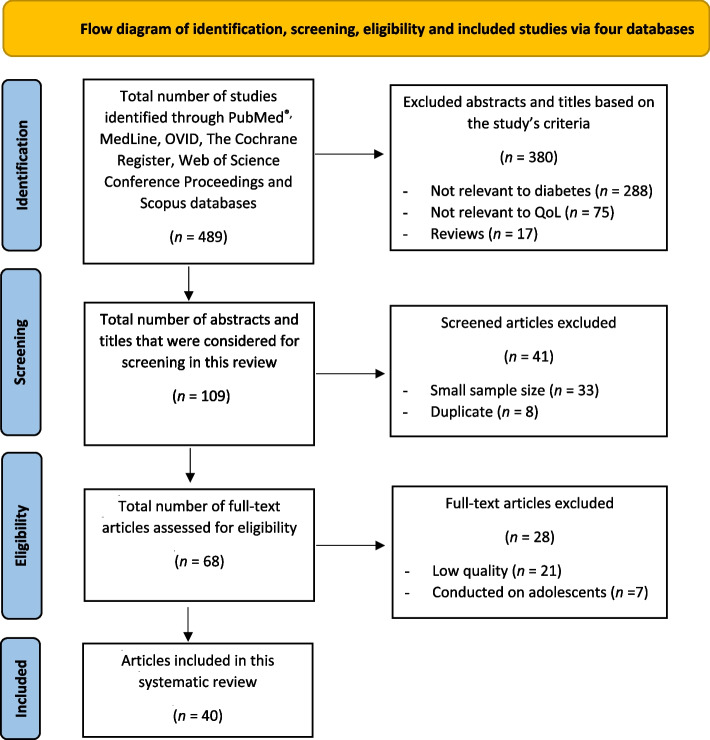
Flow diagram of identification, screening, eligibility and included studies via four databases

All studies conducted during the last decade or more (1^st^ of January 2011 to 31^st^ of July 2022) were considered to be eligible if they met the following inclusion criteria: 1) Population-based studies; 2) Among adults sharing common characteristics and health conditions including T2DM; 3) Studies focusing on health-related QoL and mental health assessment questionnaires or surveys; 4) Any studies conducted on 50 patients or more; 5) Surveys mentioned in conference abstracts were only considered if sufficient information were available for data extraction (Fig. 1). All publications were reviewed in full text to determine whether they met the inclusion criteria or not by two authors independently (Fig. 1).

### Synthesis and data extraction

According to the eligibility criteria, the main author (O.A.) carefully scanned the titles and abstracts to address any duplicated or irrelevant studies from the initial databases, PubMed, and Scopus.

This was followed by reviewing all chosen articles in their full manuscript and filling in a pre-structured table that summarises and assesses the quality of the selected studies and any general information (Table 1). The table was designed into two sections one to cover the study characteristics and the other for study quality including the following items/ categories: 1) The primary author’s name; 2) Year of publication; 3) Study location; 3) Study design; 4) Target population (included the number of participants, age, and gender); 5) Main objectives and questionnaires; 6) Mode of questionnaire administration; 7) Validity; 8) Reproducibility; 9) Responsiveness of the participants; 10) Type of bias; 11) Languages support (Table 1).

**Table 1:** Overall studies characteristics

Article characteristics						Measurement characteristics						
**S.N**	**Authors**	**Year**	**Study location**	**Study design**	**Target population**	**Questionnaires and main objectives**	**Mode of questionnaire administration**	**Validity**	**Reproducibility**	**Responsiveness of the participants**	**Type of bias**	**Languages support**
1	Wadden et al	2014	United States	Randomized clinical trial	5,145 overweight or obese adults with Type 2 Diabetes Mellitus (T2DM)	Questionnaires: The Medical Outcomes Study Short Form 36 (SF-36) and the Beck Depression Inventory (BDI) Main objectives: To assess the effects of long-term intensive lifestyle intervention on depression symptoms and Quality of Life (QoL) in patients with T2DM	Self-administered	Yes	Yes	40% for QoL at the last year of the study	Self-report and non-response bias	Yes
2	Hajos, T.R.S. et al	2013	Netherlands	Randomized clinical trial	2,055 adult patients with Type 1 Diabetes Mellitus (T1DM) or T2DM	Questionnaires: The Problem Areas in Diabetes scale (PAID), Medical Outcomes Short Form 12 (SF-12), the World Health Organization—Five Well-Being Index (WHO-5) and the 9-item Patient Health Questionnaire (PHQ-9) Main objectives: To evaluate the psychometric properties of the WHO-5 index in a large sample of Dutch outpatients with T1DM or T2DM	Self-administered	Yes	Yes	47% returned the first questionnaire and 38% of the initial population returned the second	Self-report and non-response bias	Yes except the PAID questionnaire
3	Green, A.J. et al	2012	United States	Cross-sectional	2,718 US adult households with T2DM	Questionnaires: The SF-12 and the PHQ-9 Main objectives: To examine the association of hypoglycemia with QoL and depression among adults with T2DM	Self-administered	Yes	Yes	71% from the 2008 annual follow-up Study to Help Improve Early evaluation and management of risk factors Leading to Diabetes (SHIELD) survey	Self-report and non-response bias	Yes
4	Schunk, M. et al	2012	German	Cross-sectional	846 adults between 45 and 74 years old with T2DM	Questionnaires: The SF-36 and SF-12 Main objectives: To compare the population values of QoL among patients with and without T2DM, across several large population-based survey studies from different regions in Germany and a nationwide survey	Self-administered	Yes	Yes	Overall response rates ranged between 61 and 69% from national and four regional population-based surveys (KORA, CARLA, SHIP and DHS) and the primary data which is the Diabetes Collaborative Research of Epidemiologic Studies (DIAB-CORE)	Self-report and non-response bias	Yes
5	Mazhar, K. et al	2011	United States	Cohort	1,064 above 40 years old adults with T2DM	Questionnaires: The SF-12 and the National Eye Institute Vision- Specific Questionnaire (NEI-VFQ-25) Main objectives: To evaluate the relationship between diabetic retinopathy and its severity on QoL in a population-based sample of Latinos with T2DM	Interviewer- administered	Yes	Yes	84% among the diabetic participants from the Los Angeles Latino Eye Study (LALES)	None	Yes
6	Siersma V. et al	2013	Denmark	Cross-sectional	1,232 adult patients with T2DM	Questionnaires: The EuroQoL EQ-5D Main objectives: To investigate factors determining clinical outcome, healthcare consumption and QoL in patients with new foot ulcers	Self-administered	Yes	Yes	88.30%	Self-report	Yes
7	Nicolucci, A. et al	2012	Italy	Randomized clinical trial	606 sedentary adult patients with T2DM. From the Italian Diabetes and Exercise Study (IDES)	Questionnaires: The SF-36 Main objectives: To assess the relationship between changes in QoL and volume of physical activity/exercise, in T2DM patients	Self-administered	Yes	Yes	87.6% from the 691 assessed for eligibility in this study	Self-report and non-response bias	Yes
8	Williams, E.D. et al	2012	Australia	Randomized clinical trial	120 adult participants with T2DM	Questionnaires: The SF-36 Main objectives: To evaluate the Telephone-Linked Care (TLC) Australian program designed to improve the T2DM management and QoL among the participants compared with a large Australian population study	Self-administered	Yes	Yes	92.5% of the total sample completed the six-month assessment	Self-report and selection bias	Yes
9	Pintaudi, B. et al	2015	Italy	Cross-sectional	2,374 adults with T2DM from the benchmarking network for clinical and humanistic outcomes in diabetes (BENCH-D) study	Questionnaires: The SF12, the WHO-5, Diabetes Empowerment Scale-Short Form (DES-SF), Patient Assessment of Chronic Illness Care-Short Form (PACIC-SF), Health Care Climate-Short Form (HCC-SF), Global Satisfaction with Diabetes Treatment (GSDT), Summary of Diabetes Self-Care Activities measure (SDSCA-6), Barriers to Medications (BM) and Perceived Social Support (PSS) Main objectives: To evaluate correlates of diabetes related distress in the context of the large sample of people with T2DM participating in the BENCH-D study	Self-administered	Yes	Yes	Not mentioned	Self-report bias	Yes all the instruments, with the only exceptions of the WHO-5 and SF- 12, already available in Italian language
10	Löndahl, M. et al	2011	Sweden	Randomized clinical trial	75 adults with T2DM	Questionnaires: The SF-36 Main objectives: To evaluate whether hyperbaric oxygen therapy improves QoL in these patients or not	Self-administered	Yes	Yes	98% the article mentioned that there was only two patients did not fill out the SF-36 at 12 month follow-up due to their deteriorated medical condition	Self-report bias	Yes
11	Adriaanse, M.C. et al	2016	Netherlands	Cross-sectional	1,676 with T2DM adult patients, aged between 31 and 96 years old	Questionnaires: The SF-12 Main objectives: To study the prevalence, impact and the dose–response relationship of comorbid chronic conditions on QoL in T2DM patients	Self-administered	Yes	Yes	44% from the original data derived from two data sources	Self-report bias	Yes
12	Myers, V.H. et al	2013	United States	Randomized clinical trial	212 sedentary adults with T2DM aged between 30 and 75 years old	Questionnaires: The SF-36 Main objectives: To compare the effects of aerobic, resistance, or a combination of both on QoL in sedentary individuals with T2DM	Self-administered	Yes	Yes	70% who met a minimum criteria of attendance to their exercise prescription for at least 6 months and had SF-36 data at baseline and follow-up	Self-report and non-response bias	Yes
13	Chew, B.-H. et al	2015	Malaysia	Cross-sectional	752 adults with T2DM above 30 years old	Questionnaires: The World Health Organization Quality of Life-Brief (WHOQOL-BREF), the 17-items Diabetes Distress Scale (DDS-17), and the PHQ-9 Main objectives: To examine the effects of diabetes-related distress on QoL among patients with T2DM who received regular primary medical care in three public health clinics	Self-administered	Yes	Yes	93.10%	Self-report bias	Yes
14	Shi, L. et al	2014	United States	Cross-sectional	3,999 adult patients with T2DM	Questionnaires: The EuroQoL EQ-5D and the SF-12 Main objectives: To test whether fear of hypoglycemia is independently associated with poorer QoL among patients with T2DM or not	Self-administered	Yes	Yes	20.30%	Self-report and non-response bias	Yes
15	Kuznetsov, L. et al	2014	United Kingdom	Cross-sectional	1,876 adults with T2DM aged between 40 and 69 years old	Questionnaires: The SF-36 and the Audit of Diabetes Dependent Quality of Life (ADDQoL19) Main objectives: To examine the association between health status, diabetes-specific QoL and glycemic control among individuals with T2DM	Self-administered	Yes	Yes	66% of the 2859 patients still alive at 5 years from the ADDITION-Europe trial cohort	Self-report, recall and social desirability bias	Yes
16	Bourdel-Marchasson, I. et al	2013	France	Cross-sectional	2,832 patients with T2DM adults (18 years and older)	Questionnaires: The SF-12 Main objectives: To assess QoL in people with T2DM and to estimate the relative contributions of socio-demographic factors, diabetes characteristics, complications and treatment, social support and functional impairment in daily living, in mental and physical components of QoL	Self-administered	Yes	Yes	59%	Self-report and non-response bias	Yes
17	Freemantle, N. et al	2013	United Kingdom	Randomized clinical trial	1,922 adults with T2DM from three randomized clinical trials	Questionnaires: The SF-36 Main objectives: To compare the effect of insulin degludec and insulin glargine on QoL in patients with T2DM starting on basal insulin, in combination with oral antidiabetic drugs	Self-administered	Yes	Yes	Not mentioned	Self-report bias	Yes
18	Kempf, K. et al	2012	Germany	Cohort	327 adults with T2DM not older than 75 years old	Questionnaires: The SF-36 and the Center for Epidemiologic Studies Depression Scale questionnaires (CES-D) Main objectives: To evaluate the impact of lifestyle intervention program on glucometabolic and QoL, with weight and HbA1c reduction as main outcome variables	Self-administered	Yes	Yes	70% from the participants who are completed the study	Self-report and non-response bias	Yes
19	Wermeling, P.R. et al	2012	Netherlands	Cross-sectional	2,086 adults with T2DM aged between 40 and 80 years old	Questionnaires: The SF-36 and EuroQoL EQ-5D Main objectives: To assess the association between the number and type of comorbidities and health status in a large sample of well-controlled T2DM in general practice	Self-administered	Yes	Yes	95% from the invited participants	Self-report and selection bias	Yes
20	Reach, G. et al	2013	France	Cross-sectional	1,933 adults above 18 with T2DM	Questionnaires: The SF-12 Main objectives: To evaluate the impact of insulin therapy on mental and physical quality QoL and patient adherence	Self-administered (Internet-based or online)	Yes	Yes	Not mentioned	Self-report bias	Yes
21	Donald, M. et al	2013	Australia	Cross-sectional	3,609 patients with T2DM aged between 18 years or older	Questionnaires: The Audit of Diabetes-Dependent Quality of Life (ADDQoL) Main objectives: To assess the diabetes-specific QoL of a large sample of patients with T2DM	Self-administered	Yes	Yes	27.3% from the invited sample of 14,439 registrants to participate	Self-report and non-response bias	Yes
22	Zurita-Cruz, J.N. et al	2018	Mexico	Cross-sectional	1,394 patients over 18 years of age with T2DM	Questionnaires: The SF-36 and the BDI Main objectives: To understand the relationship between glycemic control and patient-centered care to better determine its legitimacy as a means of improving care for patients with T2DM	Self-administered	Yes	Yes	Questionnaires that lacked an answer were returned to the patients to complete them	Self-report bias	Yes
23	Williams, J.S. et al	2016	United States	Cross-sectional	615 adults with T2DM above 18 years old	Questionnaires: The SF-12 Main objectives: To evaluate the relationship between patient-centered care, diabetes self-care, glycemic control, and QoL in a sample of adults with T2DM	Self-administered	Yes	Yes	Not mentioned	Self-report bias	Yes
24	Al Sayah, F. et al	2015	Canada	Controlled clinical trial	157 adults with T2DM above 18 years old	Questionnaires: The SF-12, the PHQ-9 and the EuroQoL EQ-5D Main objectives: To examine the longitudinal associations of inadequate health literacy with depression related and other health outcomes in patients with T2DM who had recently screened positive for depression in a clinical trial	Self-administered	Yes	Yes	71%	Self-report and non-response bias	Yes
25	Jayasinghe, U.W. et al	2013	Australian	Cross-sectional	2,181 adults with T2DM and/or hypertension/ischemic heart disease patients aged 18 years or more	Questionnaires: The SF-12 and the Chronic Illness Care (PACIC). Main objectives: To investigate the relationship between patient or general practitioners’ characteristics and QoL in a large sample of chronically-ill Australian adults from two states and the Australian Capital Territory	Self-administered	Yes	Yes	70%	Self-report and non-response bias	Yes
26	Hunger, M. et al	2014	German	Cohort	1,046 participants with T2DM aged between 55 and 74 years old	Questionnaires: The SF-12 Main objectives: To examine how changes between NGT, prediabetes and diabetes over a 7-year period are associated with change in QoL	Face-to-face interview at baseline and self-administered at follow-up	Yes	Yes	67% from the population-based German KORA (Cooperative Health Research in the region of Augsburg) study	Self-report and non-response bias	Yes
27	Sayah, F.A. et al	2016	Canada	Cohort	1,948 adults above 18 years old with T2DM	Questionnaires: The SF-12, the EuroQoL EQ-5D, and the PHQ8 Main objectives: To examine the association of health literacy (HL) with changes in QoL among patients with T2DM	Self-administered	Yes	Yes	Not mentioned	Self-report bias	Yes
28	Pawaskar, M. et al	2018	United States	Cross-sectional	3,630 participants above 18 years old with T2DM	Questionnaires: The SF-36 Main objectives: To explore the association between hypoglycemia severity and QoL	Self-administered (Internet-based or online)	Yes	Yes	Not mentioned	Self-report bias	Yes
29	Wan, E.Y.F. et al	2016	Hong Kong	Cross-sectional	1,826 adults with T2DM above 18 years old	Questionnaires: The SF-12 Main objectives: To identify the predictors for poorer QoL in Chinese patients with T2DM over time and provide a 2-year estimate of preference-based measure for cost-effectiveness analysis of primary care interventions for patients with diabetes	Interviewer-administered (By phone)	Yes	Yes	Between 75.5% and 59.7%	Non-response bias	Yes
30	Saffari, M. et al	2019	Iran	Cross-sectional	793 adults 65 years or older with T2DM	Questionnaires: The World health organization quality of life scale brief version (WHOQOL-BREF) and Diabetes-specific quality of life questionnaire module (DMQoL) Main objectives: To investigate how religiosity may affect disease-specific QoL	Self-administered	Yes	Yes	Not mentioned	Self-report bias	Yes
31	Alenzi, E.O. et al	2016	United States	Cross-sectional	1,033 adults aged over 21 years or older with DM and depression	Questionnaires: The SF-12 Main objectives: To examine the association between depression treatment and QoL measures of adults with DM and depression, comparing them to those who did not report any depression treatment	Interviewer-administered	Yes	Yes	Not mentioned	Self-report and recall bias	Yes
32	Abbatecola, A.M. et al	2015	Italy	Cross-sectional	558 older people with T2DM	Questionnaires: The SF-12 and ADDQoL Main objectives: To investigate the validity and reliability of the ADDQoL in older outpatients with T2DM and to investigate the association between the overall impact of diabetes assessed using the average weighted impact score from the ADDQoL, on improvement in glycemic control over time	Self-administered	Yes	Yes	Not mentioned	Self-report and selection bias	Yes
33	Thiel, D.M. et al	2017	Canada	Cohort	1,948 adults above 18 years old with T2DM	Questionnaires: The SF-12 and the EuroQoL EQ-5D Main objectives: To investigate the longitudinal relationship between physical activity and QoL in adults with T2DM	Self-administered	Yes	Yes	Not mentioned	Self-report bias	Yes
34	Janssen, L.M.M. et al	2020	United States	Cross-sectional	2,915 individuals aged between 40 and 75 years old with T2DM	Questionnaires: The SF-36 and the EuroQoL EQ-5D Main objectives: To investigate the associations of diabetes related complications and other social determinants with the costs related to T2DM and with the QoL of people with the disease	Self-administered	Yes	Yes	85% from the first participants in the Maastricht Study	Self-report and recall bias	Yes
35	Cai, J. et al	2018	United States	Randomized clinical trial	2,536 adults with T2DM	Questionnaires: The SF-36, the Impact of Weight on Quality of Life-Lite (IWQoLLite) and Current Health Satisfaction Questionnaire (CHES-Q) Main objectives: To evaluate the effect of treatment with canagliflozin, a sodium glucose cotransporter 2 inhibitor, compared with placebo or sitagliptin on QoL outcomes in participants with T2DM from the clinical development program	Self-administered	Yes	Yes	Ranged between 81 and 93%	Self-report bias	Yes
36	Zhao, H. et al	2020	Canada	Cohort	969 adults above 18 years old with T2DM	Questionnaires: The SF-12 and the EuroQoL EQ-5D Main objectives: To evaluate the relationship between diabetic foot disease and QoL over a 2-year period	Self-administered	Yes	Yes	82%	Self-report bias	Yes
37	Lloyd, C.E. et al	2020	Switzerland	Cohort	1,616 adults with T2DM aged between 18 and 65 years old	Questionnaires: The PHQ-9, the WHO-5 and the PAID Main objectives: To identify specific risk factors for the onset of diagnosed depression as well as depressive symptoms in this cohort of individuals with T2DM	Self-administered	Yes	Yes	Not mentioned	Self-report bias	Yes
38	Sacre, J.W. et al	2021	Australia	Cross-sectional	470 adults with T2DM aged between 18 and 80 years old	Questionnaires: The Generalised Anxiety Disorder (GAD-7), the PHQ-8, the PAID, and the Confidence in Diabetes Self-Care (CIDS) scale and 12-item Diabetes Support Scale (DSS) Main objectives: To investigate worry about COVID-19 and its perceived impact on QoL and healthcare access among adults with T2DM	Self-administered (phone and online)	Yes	Yes	96%	Self-report and selection bias	Yes
39	Selenius, J.S. et al	2020	Finland	Cross-sectional	1,930 adults with T2DM	Questionnaires: The SF-36 and the BDI Main objectives: To investigate whether the association between the different degrees of impairment in glucose regulation and QoL is modified by the severity and type of depressive symptoms	Self-administered	Yes	Yes	Not mentioned	Self-report bias	Yes
40	Nicolucci, A. et al	2021	Italy	Cross-sectional	12,028 adults with T2DM	Questionnaires: The SF-36-Item and the Hypoglycemia Fear Survey-II (HFS-II) Main objectives: To investigate factors associated with QoL in patients with T2DM at initiation of second-line glucose-lowering therapy	Self-administered	Yes	Yes	Between 69.1% and 72.6%	Self-report and non-response bias	Yes

This table was organised based on the frequency of citations (from the highly cited article to the least cited article)

A 10% random sample was checked by a second reviewer (K.H.) to check for the search and reviewing of the articles, references, and any additional relevant publications that may have been missed by the initial electronic databases was finally carried out independently by two senior examiners. Any inconsistencies were discussed by a third reviewer (J.F.) for a final decision.

### Quality appraisal

The methodological quality of each included study in terms of validity, reliability, and consistency was assessed using the Joanna Briggs Institute (JBI) critical appraisal checklists (https://jbi.global/critical-appraisal-tools) for cohort, randomized controlled trials (RCTs), and cross-sectional studies which was the most appropriate and applicable tool for this review [13]. The JBI checklist for cohort studies consists of 11 items, while 13 items for RCTs, and 8 items for cross-sectional studies. Each item was answered with either a Yes, No, Unclear, or Not Applicable response.

The categories of the studies were divided into: High quality (if 80% or more of the items were answered with a yes), Moderate (if more than 60% of the items were answered with a yes), and Low (if less than 60% of the items were answered with yes). Any study categorized as high or moderate quality was eligible to be included in this review. Any disagreement between the reviewers was solved by a discussion with the third reviewer (J.F.).

## Results

### Search and eligible studies

A total of 489 articles were identified in six electronic medical databases, 343 of which were selected (58.6% from Scopus) during the first screening (Fig. 1). Following the first screening, 109 articles were identified and subjected to the next level of screening after reading the titles and abstracts (Fig. 1). Of these, 68 articles were considered potentially eligible after reviewing the full text (Fig. 1). Subsequently, 28 articles were excluded based on the defined inclusion and exclusion criteria and there were 21 articles [14–34] among them considered as low quality and excluded based on the JBI quality appraisal checklists used in this review (Fig. 1) (Table 2). Finally, 40 articles were shown to meet our eligibility criteria and were, therefore, included in this systematic review (Fig. 1) (Table 1).

**Table 2:** Summary of quality appraisal for excluded studies

S.N	Authors	Year	Study design	Rationale for exclusion
1	Cykert, D. M., et al	2017	Cross-sectional	The exposure measured was not clearly defined in a valid and reliable way and the standard criteria used for the measurement of the outcomes was unclear
2	Rani, M., et al	2019	Cross-sectional	The study subjects and setting were not clearly described. Confounding factors were not mentioned by the authors
3	Babenko, A. Y., et al	2019	Cross-sectional	The methodology provided no details on the study subjects and setting. The study lacked details on confounding factors
4	Haidari, F., et al	2017	Cross-sectional	The standard criteria for measuring the outcomes and confounding factors were not clear or identified
5	Pati, S., et al	2020	Cross-sectional	The exposure and outcomes variables were not measured in a valid and reliable way
6	Thapa, S., et al	2019	Cross-sectional	Confounding factors and strategies used to deal with these were not identified in this study. The methodology did not provide clear details on the study participants and setting
7	Sionti, V., et al	2019	Cross-sectional	Unclear inclusion criteria, study setting, confounding factors, and statistical analysis
8	Altınok, A., et al	2016	Cross-sectional	There were no proper details on the study participant, setting, and any confounding factors
9	Mikailiūkštienė, A., et al	2013	Cross-sectional	The standard criteria for the measurement of the outcome variables were unclear and there were no details on the study subjects and setting
10	Dalal, J., et al	2020	Cross-sectional	There were no confounding factors identified. Unclear outcomes measurement and statistical analysis
11	Nyoni, A. M., et al	2018	Cross-sectional	There were no standard criteria used for measuring the outcomes variables and limited details on the study participants and setting
12	Olukotun, O., et al	2022	Cross-sectional	There were no confounding factors identified and the study setting was not clearly mentioned
13	Sato, M. and Y. Yamazaki	2012	Cross-sectional	The validity and reliability for measuring the outcomes were unclear and there were no confounding factors identified
14	Walker, R. J., et al	2014	Cross-sectional	The strategies for dealing with confounding factors as well as what was used as the standard criteria for measuring the outcomes variables were unclear
15	Baruah, M. P., et al	2021	Cross-sectional	The exposure and outcomes were not measured in a valid and reliable way. The study setting was unclear and no identification of confounding factors
16	Hu, F., et al	2015	Cross-sectional	Unclear inclusion criteria for the studied population and strategies for identifying the confounding factors
17	Hashimoto, Y., et al	2020	Cross-sectional	There were unclear inclusion criteria and no appropriate information about identifying the confounding factors
18	Abraham, A. M., et al	2020	Randomized clinical trial	There was no true randomization used for assignment of participants to treatment groups. Allocation concealment was not done
19	Kempf, K. and S. Martin	2013	Randomized clinical trial	The trial design was not appropriate and there was no detailed information about any deviations from the standard trial design accounted for the conduct and analysis of the trial
20	Ebrahimi, H., et al	2018	Randomized clinical trial	There was no information as to whether the outcomes assessors were blinded to the intervention or not. Unclear baseline similarity in the two groups. The outcome measurements were not clearly conducted in a reliable way
21	Costa, M. S. A., et al	2020	Cohort	Unclear whether the groups or the participants were free of the outcomes or not at the baseline of the study. There were no clear strategies to address the incomplete data

This table is based on the JBI quality appraisal checklists

### Study characteristics and QoL measurements

The majority of the studies were cross-sectional 60% [35–58], followed by 22.5% clinical trial [59–67], and 17.5% cohort [68–74]; with overall response rates ranging between 40 and 98% among adult patients with T2DM.

The following questionnaires used in the QoL assessment included the Medical Outcomes Study Short Form 36 (SF-36), the Medical Outcomes Short Form 12 (SF-12), the 9-item Patient Health Questionnaire (PHQ-9), the EuroQoL EQ-5D, The World Health Organization Quality of Life-Brief (WHOQOL-BREF), the 17-items Diabetes Distress Scale (DDS-17), the Audit of Diabetes Dependent Quality of Life (ADDQoL19), the Diabetes‑Specific Quality of Life (DMQoL), and the Impact of Weight on Quality of Life-Lite (IWQoLLite). Other questionnaires used evaluated the mental health combined with QoL assessment. This included the Beck Depression Inventory, the World Health Organisation—Five Well-Being Index (WHO-5), the Chronic Illness Care (PACIC), the Center for Epidemiologic Studies Depression Scale questionnaires (CES-D), the Generalised Anxiety Disorder (GAD-7), the Problem Areas in Diabetes (PAID) scale, the Confidence in Diabetes Self-Care (CIDS) scale, the 12-item Diabetes Support Scale (DSS), the Hypoglycaemia Fear Survey-II (HFS-II), the Health Care Climate-Short Form (HCC-SF), the Global Satisfaction with Diabetes Treatment (GSDT), the Summary of Diabetes Self-Care Activities measure (SDSCA-6), the Barriers to Medications (BM), the Perceived Social Support (PSS), and The Empowerment Scale-Short Form (DES-SF).

### Main findings

The six top commonly used QoL measurements included the SF-12 which was found in 19 studies [35, 36, 38, 39, 41, 43, 45, 48, 49, 51, 53, 54, 60, 66, 68, 70–73], the SF-36, identified in 16 studies [36, 42, 44, 47, 50, 55, 57–59, 61–65, 67, 69], the EuroQoL EQ-5D, included in 8 studies [37, 41, 44, 55, 60, 71–73], the PHQ-9, found in five studies [35, 40, 60, 66, 74], the WHOQOL-BREF, evaluated in two studies [40, 52], and the ADDQoL19, identified in two studies [42, 46].

Fifteen (37.5%) studies used only one questionnaire. In this regard, the SF-12, was used as a single questionnaire in seven studies [39, 43, 45, 48, 51, 53, 70], the SF-36 in six studies [50, 61–65], the EuroQoL EQ-5D in one study [37] and the ADDQoL19 in one study [46]. However, the remaining reviewed studies (62.5%) used more than one questionnaire.

In terms of mental health measurements, there were four questionnaires that were commonly used which combined with QoL questionnaires namely the WHO-5 in three of the reviewed studies [38, 66, 74], the BDI in three studies [47, 57, 67], the PAID in three studies [56, 66, 74], and lastly the PACIC, found in two studies [38, 49].

Most of the studies (90%) reported using self-administered questionnaires with only four [51, 53, 68, 70] identified to use interviewer mode of administration. Moreover, all of the studies indicated that the questionnaires used were validated, reliable and that they supported different languages.

## Discussion

The present systematic review indicates that the SF-12 questionnaire is the most appropriate and commonly used measurement to assess QoL and mental health followed by the SF-36, the EuroQoL EQ-5D, the PHQ-9, the WHOQOL-BREF, and the ADDQoL19. This questionnaire was used in several studies with different methodological approaches and was confirmed to be validated, reliable, less time-consuming, easy to use and available in many languages [75]. Other attributes of the SF-12 questionnaire include that it is a self-administered generic measurement and large-scale, population-based health inventory that has been developed to measure both the physical and mental health aspects of a patient [75]. It is effective and efficient with a completion time of fewer than five minutes [75]. Moreover, it has the exact eight health domains (Physical Functioning, Role Physical, Role Emotional, Mental Health, Bodily Pain, General Health, Vitality, and Social Functioning) similar to SF-36 but with one or two items per domain and without any notable statistical difference especially for studies with a large sample size [75]. These were the significant advantages of using SF-12 over SF-36 while the disadvantages were considered as less in represents or comprehensiveness of the content of health measures and lacking of the statistical precision of mental and physical components scores compared to SF-36 [75].

One of the largest randomized controlled trials (RCTs) titled Look AHEAD (Action for Health in Diabetes) conducted on 5,145 overweight or obese with T2DM assessed the effect of long-term lifestyle modification on QoL and depression symptoms using the BDI and SF-36 questionnaires as the main measurement for their primary outcomes. Concerns included a shallow response rate by fewer than 40% of patients in the final year of the study possibly due to the high dropout rate and lengthy QoL questionnaire [67]. Another RCT was conducted among 1,922 patients with T2DM to evaluate the effect of two different insulin therapy on QoL using the SF-36 alone. The authors of this study observed that there was a lack of a sleep variable on the questionnaire which was considered as a study limitation. There was no information relating to the response rate in this study [61]. The remaining trials that were included in the present review used the SF-36 with a response rate between 70%-98%; with the exception of one controlled clinical trial that used the SF-12 combined with different questionnaires and most of which had weaknesses with respect to randomization, blinding, and allocation concealment [59, 60, 62–66].

Another population-based cohort study on adults with T2DM conducted on 1,064 participants to assess the impact of diabetic retinopathy on QoL used the SF-12 where interviewers had the questionnaire administered in either English or another language [68]. This was similar to a population-based German cohort study that used the SF-12 to examine the change of QoL in 1,046 diabetic patients through a face-to-face questionnaire administered at baseline where the response rate was between 67 to 84% [70]. However, most of the other cohort studies included in this review preferred to use the SF-12 as a main questionnaire for their studies [71–73].

A longitudinal cross-sectional study conducted to identify the determinants of poor QoL in 1,826 Chinese diabetic patients who used the SF-12 over 24 months (through a phone interview) had a response rate between 75.5% and 59.7% [51]. This study used a similar methodological approach with another longitudinal cross-sectional study regarding the association between depression and QoL among 1,033 adults with T2DM addressed by interviews throughout the study using the SF-12 questionnaire alone [53]. It has been plausible that the majority of the cross-sectional studies matched with cohort studies in terms of using the SF-12 as their primary questionnaire and through interview mood of administration [35, 36, 38, 39, 41, 43, 45, 48, 49, 54].

### Strengthens and limitations

The main strength of this review is that we comprehensively reviewed the body of evidence that focused on the most common and widely used publications over the last decade. This study identified the most common, widely used efficient and validated QoL and mental health questionnaire over a large number of publications for more than a decade in different languages. There are some weaknesses due to potential biases identified from the included studies especially the self-reported and non-response bias as well as the differences in response rates. Another weakness is the lack of standard terminology which may possibly cause misleading results. Lastly, the huge heterogeneity in the study designs, methodology, and sample size has limited our ability to quantify any differences through a meta-analysis.

## Conclusion

In the backdrop of the growing prevalence of this disease worldwide there has been limited information on the most efficient and commonly used questionnaire for the diabetic patient. Our review found evidence of the effects of six different QoL and mental health questionnaires. Findings identified the SF-12 as the most validated, time efficient and effective questionnaire that allows cross-culture adaption which can be used in population-based studies across the world. These results encourage the use of SF-12 in adult patients with T2DM as a useful screening measure for identifying and monitoring mental health issues that may assist with target treatment and prevention. The wide range of tools used to assess QoL, methodology of administration, clinical research question and limited sample size used by studies hinder direct comparisons in patients with T2DM. Future large multicentre prospective research is recommended to help clarify causality on associations between mental health, QoL and any barriers in people with T2DM involving individuals from different cultural backgrounds.

## Data Availability

Not applicable.

## Abbreviations

T2DM: Type 2 diabetes mellitus
QoL: Quality of Life
SF-12: Medical outcomes short form 12
SF-36: Medical outcomes short form 36
